# Clip-and-pull method: re-strangulation of a sizable small-bowel polyp for endoscopic ischemic polypectomy

**DOI:** 10.1055/a-2308-8176

**Published:** 2024-05-17

**Authors:** Tomoko Tamaru, Kunihiko Oguro, Tomonori Yano, Yusuke Ono, Hirotsugu Sakamoto, Edward J Despott, Hironori Yamamoto

**Affiliations:** 1 12838 Department of Medicine, Division of Gastroenterology, Jichi Medical University, Shimotsuke, Japan; 2 171090 Royal Free Unit for Endoscopy, The Royal Free Hospital, University College London Institute for Liver and Digestive Health, London, United Kingdom of Great Britain and Northern Ireland

Double-balloon endoscopy (DBE) facilitates safe endotherapy of small-bowel polyps 1 . Endoscopic ischemic polypectomy (EIP) with detachable snares or clips is useful as a secure and safe method for treating benign polyps 2 3 4 , especially in patients with Peutz–Jeghers syndrome, who require polyp management to avoid intussusception 5 . However, EIP with detachable snares in the small bowel may be technically challenging. Furthermore, initial strangulation and resultant shrinkage of the lesion may loosen the ligature, and this may result in incomplete ischemia. In this situation, re-strangulation is required.

However, it is often difficult to perform re-strangulation by hooking the loop of the detachable snare and pulling it into the sheath ( Fig. 1 ). Considering this, we have developed a new, easier method for re-strangulation with a detachable-snare: the “clip-and-pull” method ( Fig. 2 ).

**Fig. 1:**
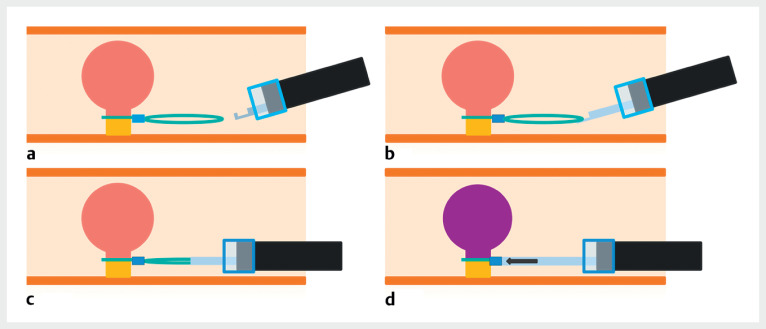
Conventional method for re-strangulation using a detachable snare. **a, b** The loop of the snare is caught with the hook. **c** The loop is pulled into the sheath. **d** The sheath pushes a stopper.

**Fig. 2:**
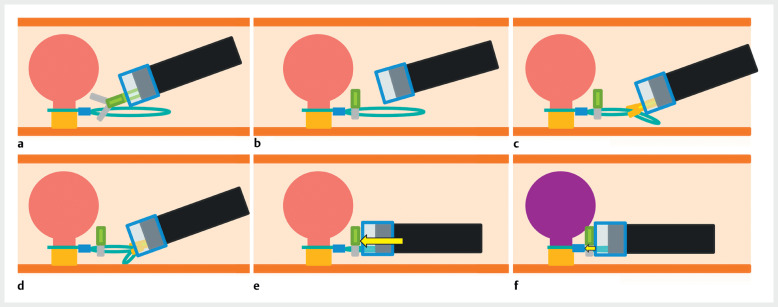
The “clip-and-pull” method. **a, b** A clip is placed on the free part of the loop. **c** The distal side of the loop is grasped with a grasper. **d** The loop is pulled into the instrument channel. **e** The enteroscope pushes the clip. **f** The clip in turn slides the stopper along the loop, further tightening the detachable snare.

A 45-year-old woman with Peutz–Jeghers syndrome was found to be suffering from polyp-related intussusception of the terminal ileum into the cecum ( Fig. 3 ).

**Fig. 3:**
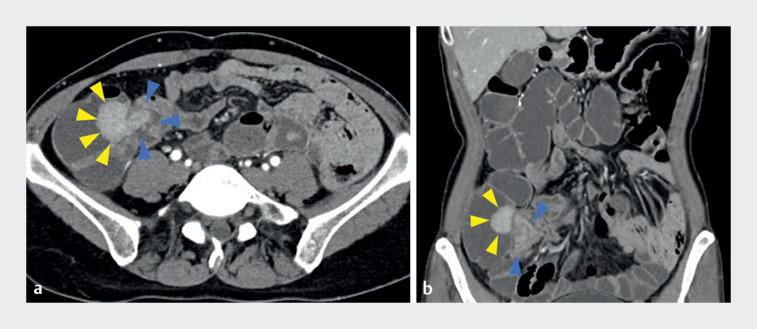
Computed tomography (CT) images of intussusception of the terminal ileum into the cecum (blue arrowheads) related to the sizable polyp (yellow arrowheads). **a** Axial view. **b** Coronal view.

We first performed retrograde DBE for EIP of the culprit polyp using a detachable snare. We placed a detachable snare on the stalk of the polyp, pushed the polyp into the terminal ileum using the enteroscope, and strangulated the stalk. We then inserted the enteroscope further up to the mid-ileum to treat any other polyps. On withdrawal of the enteroscope into the terminal ileum, the culprit polyp was noted to be insufficiently discolored. We therefore placed a long-type clip onto the free part of the loop of the detachable snare, grasped the distal side of the loop with a grasper, and pulled it into the instrument channel. Through this maneuver, the enteroscope pushed the clip, which in turn slid the loop stopper along the loop to further tighten the detachable snare ( Video 1 ). This led to adequate ischemia as evidenced by purple discoloration of the polyp.

The “clip-and-pull” method, used in a case of re-strangulation of a polyp in a patient with Peutz–Jeghers syndrome. EIP, endoscopic ischemic polypectomy; DBE, double-balloon enteroscopy.Video 1

The clip-and-pull method is simple and useful for sizable polyps that require additional treatment after initial strangulation with a detachable snare.

Endoscopy_UCTN_Code_TTT_1AP_2AD

## Notes

Endoscopy E-Videos https://eref.thieme.de/e-videos
*E-Videos* is an open access online section of the journal *Endoscopy* , reporting on interesting cases and new techniques in gastroenterological endoscopy. All papers include a high-quality video and are published with a Creative Commons CC-BY license. Endoscopy E-Videos qualify for HINARI discounts and waivers and eligibility is automatically checked during the submission process. We grant 100% waivers to articles whose corresponding authors are based in Group A countries and 50% waivers to those who are based in Group B countries as classified by Research4Life (see: https://www.research4life.org/access/eligibility/ ). This section has its own submission website at https://mc.manuscriptcentral.com/e-videos .
